# Serum zonulin as a marker of intestinal mucosal barrier function: May not be what it seems

**DOI:** 10.1371/journal.pone.0210728

**Published:** 2019-01-14

**Authors:** Mary Ajamian, David Steer, Gennaro Rosella, Peter R. Gibson

**Affiliations:** 1 Department of Gastroenterology, Monash University, The Alfred Hospital, Melbourne, Victoria, Australia; 2 Monash Biomedical Proteomics Facility, Clayton, Victoria, Australia; Consiglio Nazionale delle Ricerche, ITALY

## Abstract

The protein, zonulin, has emerged as a popular serological marker to assess the integrity of the intestinal mucosal barrier. However, there is limited information on the utility of serum zonulin to indicate gastrointestinal disease and the validity of zonulin detection in widely-used commercial assays. The current study reports differences in zonulin levels across patient groups with gastrointestinal dysfunction compared with healthy individuals, though methodological inconsistencies indicated that actual zonulin protein was not detected by the commercial assays applied. The nature of the assays’ detected antigen was investigated using immunoprecipitation followed by mass spectrometric analysis and sodium dodecyl sulphate-polyacrylamide gel electrophoresis (SDS-PAGE) followed by protein staining. Top matches of the assays’ detected antigen included haptoglobin and complement C3 for the assay manufactured by CUSABIO (Wuhan, China) and complement C3 for the assay manufactured by Immundiagnostik AG (Bensheim, Germany). These findings confirm that current commercial zonulin assays are not detecting the actual protein as prehaptoglobin-2. Until assay methodology is improved, we advise the greater scientific and medical community to exercise caution in considering the measurement of serum zonulin as a marker of mucosal barrier integrity.

## Introduction

Key roles of the intestinal epithelium are to regulate solute and fluid exchange as well as absorb nutrients [1]. An increasing number of studies point to an additional role of the gut epithelium and associated structures, along with gut-associated lymphoid tissue and the neuroendocrine network, as regulators in the passage of environmental antigens from the intestinal lumen into the sub-mucosa [2]. According to the proposed paradigm, dysregulation of the mucosal barrier leads to the increased passage of antigens and other macromolecules from the external environment into the host and initiates local and/or systemic inflammation and immune activation; this process attributed to “gut leakiness” influences tolerance and immunity.

Antigens and macromolecules may pass through the epithelial barrier via transcellular or paracellular pathways. The transcellular pathway usually involves the action of passive or active transport channels for specific substrates [3]. The paracellular pathway filters by charge and size, and is a key route of entry for macromolecules [3, 4]. Tight junctions between intestinal epithelial cells are directly implicated in the paracellular route and establish a concentration gradient that is important for both transcellular and paracellular transport [3, 4]. As such, these structures are the rate-limiting step in transepithelial transport and are central determinants of mucosal permeability [3].

The protein, zonulin, is capable of reversible tight junction disassembly and, therefore, is implicated in the regulation of mucosal permeability [5, 6]. Zonulin was first discovered as an endogenous human analogue of the bacterial enterotoxin, zonula occludens toxin (Zot), which is produced by the intestinal bacterium *Vibrio cholera* [6, 7]. To initiate tight junction disassembly, it is proposed that zonulin activates epidermal growth factor receptor (EGFR) through proteinase activated receptor 2 (PAR_2_) as well as G protein-coupled receptor PAR_2_, which transactivates EGFR [5, 8]. Activation of these two receptors decreases transepithelial electrical resistance, therefore implicating increased intestinal permeability [5]. The observation that Zot activates intracellular cascades that lead to protein kinase C α-mediated actin polymerization suggests that cytoskeleton modulation is involved in enhancing intestinal permeability [9]. As Zot and zonulin are analogues, a similar mechanism of activation associated with zonulin is suspected.

As the precursor to haptoglobin-2, zonulin belongs to the haptoglobin family of proteins. Haptoglobins are acute-phase reaction proteins that have a primary role in haemoglobin scavenging, in which they form a complex with haemoglobin to prevent oxidative damage to the haemoglobin itself and surrounding tissues [10, 11]. Haptoglobins also exert angiogenic and immunomodulatory properties [11]. Three genetic polymorphisms in human haptoglobin expression, Hp1-1, Hp2-1, and Hp2-2, are determined by the *HP1* and *HP2* alleles harboured by chromosome 16q22 [11]. As zonulin is the precursor to haptoglobin-2, individuals who bear the heterozygous Hp2-1 or homozygous Hp2-2 polymorphism are zonulin-producers whereas those with the homozygous Hp1-1 polymorphism are unable to produce zonulin. Dimerisation of haptoglobins occurs cotranslationally and proteolytic cleavage from precursor to active forms takes place while still in the endoplasmic reticulum [12]. As such, the endoplasmic reticulum contains the highest amounts of zonulin as uncleaved, pre-Hp2, yet zonulin has been reported to be measured extracellularly and detectable in human sera [5].

Dysregulation of the zonulin pathway and subsequent “gut leakiness” due to increased intestinal permeability has been associated with the pathogenesis of gastrointestinal disorders such as coeliac disease, non-coeliac wheat sensitivity/irritable bowel syndrome (NCWS/IBS), and inflammatory bowel disease (IBD) [5, 13–15]. Autoimmune, inflammatory and neoplastic diseases have also been implicated [14]. To date, there has been one conference proceeding that sought to characterise circulating levels of zonulin in NCWS, IBS and coeliac disease, and found significantly higher levels in all patient groups compared to healthy individuals (p<0.001) using a commercial assay manufactured by CUSABIO (Wuhan, China) [16]. Despite the limited evidence, commercial zonulin assays have been widely used as a convenient method to assess intestinal permeability in a variety of clinical conditions beyond gastrointestinal disease. In a recent PubMed and Google Scholar search, 10 publications used the commercial assay by CUSABIO and 61 publications used a commercial assay by Immundiagnostik AG (Bensheim, Germany) to detect human plasma or serum levels of zonulin (S1 Table).

Zonulin has received considerable attention for its potential involvement in the pathogenesis of gastrointestinal disease and candidacy as a biomarker of intestinal barrier dysfunction, yet the strength of evidence that it is a specific, reliable serum marker of disease has yet to be examined. In the present study, the primary aim was to measure serum zonulin levels in patients with well-characterised NCWS, coeliac disease, and acute severe ulcerative colitis as well as healthy controls using commercial zonulin assays. However, due to methodological shortcomings in these assays, we sought to determine whether the assays are reliably detecting zonulin as prehaptoglobin-2 and if not, what they may be detecting instead. To accomplish this, we conducted immunoprecipitation experiments followed by mass spectrometric analysis and SDS-PAGE followed by protein staining. The results from our study resolve whether serum zonulin, as measured by current commercial assays, is a useful marker of gastrointestinal dysfunction and mucosal barrier integrity.

## Materials and methods

### Patients and controls

Serum samples were collected from well-characterised patients and healthy individuals between the ages of 16 and 70 years living in Melbourne, Australia. Samples from the patient (i.e. non-coeliac wheat sensitivity, coeliac disease, and ulcerative colitis) cohorts were de-identified and featured in previous studies, which have been peer-reviewed and published [17–19]. Samples selected for testing were from patients at baseline conditions prior to any dietary or drug interventions in respective studies. Healthy subjects (n = 49) between the ages of 18 and 65 years were recruited from online and local advertisements. Exclusion criteria were allergy or sensitivity to gluten, adherence to a gluten-free diet, evidence of gastrointestinal disease, active infection or inflammation, immune abnormalities, diabetes, or liver disease. Serum from peripheral blood samples were stored at -80°C to maintain stability. All study participants gave written, informed consent and the protocols were approved by the Monash University Human Research Ethics Committee (Melbourne, Australia).

### Determination of haptoglobin phenotype

A protocol to determine the haptoglobin phenotypes of study participants by immunoblotting was adapted from information generously provided by Dr Alessio Fasano and Craig Sturgeon of the Harvard Medical School Celiac Research Program (Boston, MA, USA). Serum proteins were separated by SDS-PAGE using the Novex Mini Gel Tank electrophoresis system (ThermoScientific, Carlsbad, CA, USA) followed by dry transfer to polyvinylidene difluoride (PVDF) membranes for use in immunoblotting. To prepare proteins for separation by SDS-PAGE, 1 μL of neat serum and approximately 5 μg of protein standards for human haptoglobin phenotypes 1–1 and 2–2 (Sigma-Aldrich, St. Louis, MO, USA) were combined with 2-mercaptoethanol and Novex Tris-Glycine SDS Sample Buffer (Thermoscientific, Carlsbad, CA, USA), heated at 100°C for 10 minutes, then added to wells of 16% Tris-Glycine Mini Gels (ThermoScientific, Carlsbad, CA, USA). Proteins were then transferred to PVDF membranes by the iBlot2 gel transfer device (ThermoScientific, Carlsbad, CA, USA). Membranes were then blocked for 1 hour at room temperature or 4°C overnight in 5% bovine serum albumin in Tris-buffered saline with 0.05% Tween-20 (TBS-T). After blocking, mouse anti-human haptoglobin primary antibody, clone 26E11 (AbFrontier, Seoul, South Korea) at a 1:1000 dilution in blocking solution was added to membranes and left to incubate for 2 hours at room temperature or 4°C overnight. Membranes were then washed in TBS-T then incubated with fluorescent goat anti-mouse IgG (H+L) secondary antibody (ThermoScientific, Carlsbad, CA, USA) at a 1:5000 dilution in the dark for 30 minutes at room temperature. Membranes were then washed and imaged at 700 nm by the Li-Cor/Odyssey infrared image system (Li-Cor Biosciences, Lincoln, NE, USA).

### Commercial zonulin assays

Concentrations of zonulin in the sera were determined by commercially-available ELISA assays according to the manufacturers’ protocols from CUSABIO (Wuhan, China) and Immundiagnostik AG (Bensheim, Germany). All study samples and standards were tested in duplicate.

### Recombinant zonulin protein manufacturing and acquisition

A recombinant zonulin protein sequence from a foundational study confirming the identity of zonulin as prehaptoglobin-2 was generously provided by Dr. Alessio Fasano [5]. Recombinant protein was produced by GenScript (Piscataway, NJ, USA) using the pFastBac1 Bac-to-Bac Baculovirus Expression System.

### Immunoprecipitation of serum antigens bound to commercial kit antibodies

To collect the target antigens of CUSABIO and Immundiagnostik zonulin commercial assays, protocols for the immunoprecipitation of the serum antigen-immobilised antibody complex were developed as previously described with modifications [20]. Undiluted serum samples with high purported zonulin levels, as determined by each respective commercial assay, were selected as antigen sources. Recombinant zonulin was selected as a positive control. Immunoprecipitation buffer, i.e. Novex Tris-Glycine SDS Sample Buffer with 2-mercaptoethanol, was selected as a negative control. Biotinylated zonulin tracer, which competes with serum antigen in binding to immobilised plate antibodies of the Immundiagnostik assay, was also subject to the immunoprecipitation protocol to determine any potential interactions.

### Trypsin digestion with reduction and alkylation of immunoprecipitation products and assay standards

Immunoprecipitation samples were then prepared for comparative mass spectrometry analysis. CUSABIO assay standard, Immundiagnostik tracer, and recombinant zonulin were assay standards selected for analysis. Samples and standards were reduced in 2.5mM DTT at 50°C for 30 minutes followed by alkylation with 10mM iodoacetamide for 30 minutes in the dark at room temperature. Following alkylation, a solution containing 1 μg trypsin (Promega Corp., Madison, WI, USA) in 20mM ammonium bicarbonate (pH 7.8) was added and the samples incubated at 37°C overnight.

### Mass spectrometric acquisition

Tryptic digests were analysed by LC-MS/MS using the QExactive mass spectrometer (Thermo Scientific, Bremen, Germany) coupled online with a RSLC nano HPLC (Ultimate 3000, Thermo Scientific, Bremen, Germany). Samples were concentrated on a 100 μm, 2 cm nanoviper pepmap100 trap column with 98% buffer A (0.1% Formic acid) at a flow rate of 15 μL/minute. The peptides then eluted and separated with a 50 cm Thermo RSLC pepmap100, 75 μm id, 100Ǻ pore size, reversed phase nano column starting with 97.5% buffer A (0.1% formic acid) to 40% B (80% acetonitrile 0.1% formic acid) over a 30 minute gradient, at a flow rate of 250 nL/minute. The eluant was nebulised and ionised using the Thermo nano electrospray source with a distal coated fused silica emitter (New Objective, Woburn, MA, USA) with a capillary voltage of 1900V. Peptides were selected for MS/MS analysis in Full MS/dd-MS^2^ (TopN) mode with the following parameter settings: TopN 10, resolution 17500, MSMS AGC target 1e5, 120ms Max IT, NCE 27 and 2 m/z isolation window.

Data from LC-MS/MS run was exported to Mascot generic file format (*.mgf) using proteowizard 3.0.3631 (open source software, http://proteowizard.sourceforge.net) and searched against Swiss-Prot databases using the MASCOT search engine (version 2.4, Matrix Science Inc., London, UK) with all taxonomy selected. The following search parameters were used: missed cleavages, 1; peptide mass tolerance, ± 10 ppm Da; peptide fragment tolerance, ± 0.02 Da; peptide charge, 2+, 3+ and 4+; fixed modifications, carbamidomethyl; variable modification, oxidation (Met).

### SDS-PAGE of immunoprecipitation products and gel staining

Proteins in immunoprecipitation samples were separated by SDS-PAGE and visualised by gel staining for comparison to protein standards that appeared as top matches in mass spectrometry analysis. Immunoprecipitation samples, 5 μg of haptoglobin standards, and 5 μg of complement C3c standard (Athens Research and Technology, Athens, GA, USA) were combined with 2-mercaptoethanol, heated to 100°C for 10 minutes, then added to wells of 8–16% Tris-Glycine Mini Gels (ThermoScientific, Carlsbad, CA, USA). Proteins were separated by SDS-PAGE using the Novex Mini Gel Tank electrophoresis system, then stained with Pierce Silver Stain (ThermoScientific, Carlsbad, CA, USA) for immunoprecipitation products or Invitrogen SimplyBlue SafeStain (ThermoScientific, CA, USA), a Coomassie-based stain, for standards.

### Data analysis

Statistical analyses were performed by IBM SPSS Statistics Version 24 (IBM Corp., Armonk, NY, USA) and GraphPad Prism 6 (GraphPad Software, La Jolla, CA, USA). Figures were generated with GraphPad Prism 6. Tests for normality were determined by Shapiro-Wilk tests. Differences in purported zonulin levels between study cohorts were evaluated by Mann-Whitney U tests for nonparametric distributions. Comparative analyses between assays were performed using Spearman’s r and Bland-Altman plots. All p-values were two sided and determined to be statistically significant at p≤0.05.

## Results

### Demographics of study participants and haptoglobin phenotyping

**Fig 1:**
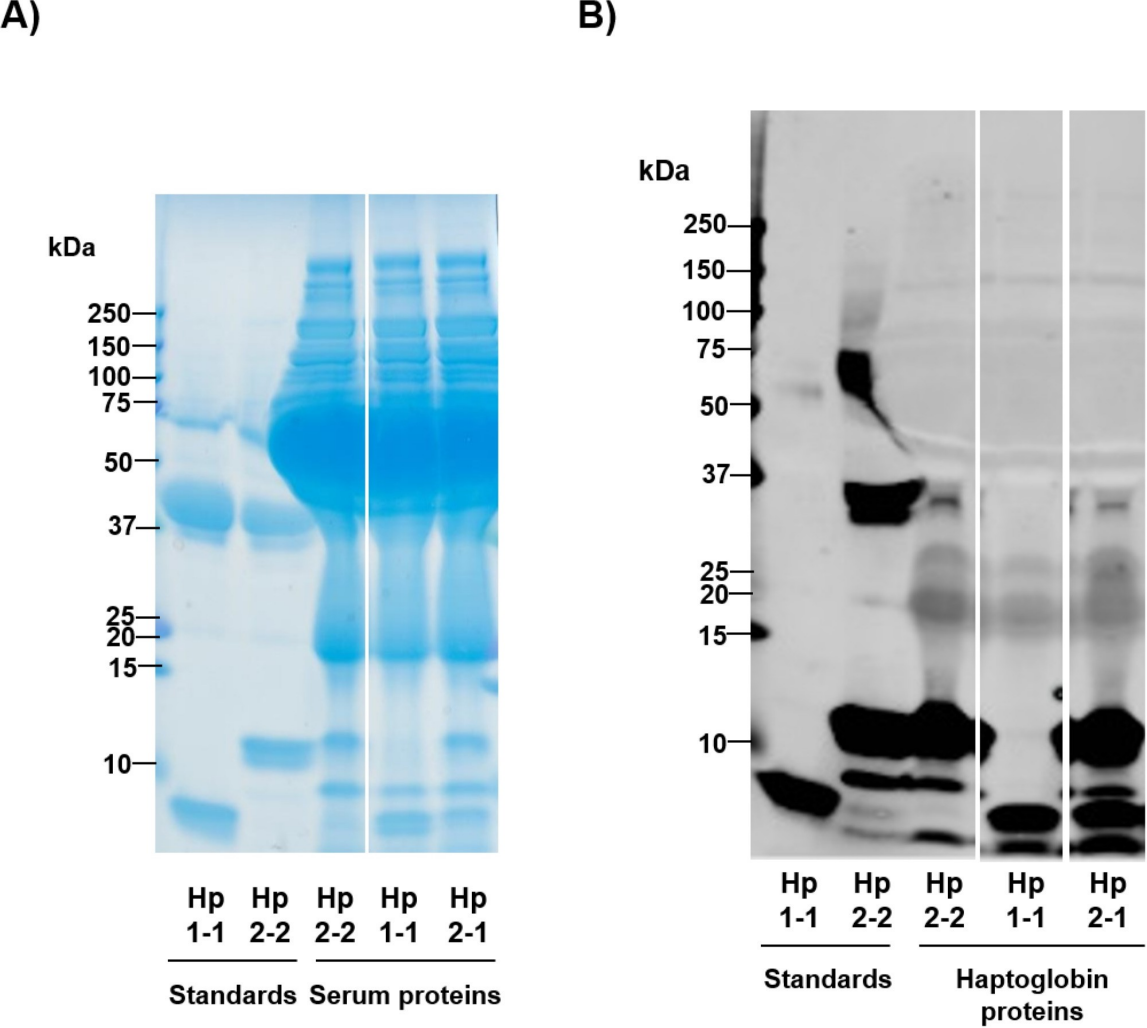
Haptoglobin phenotyping analysis by immunoblot. Haptoglobin proteins in serum samples of patients and controls were detected by anti-haptoglobin polyclonal antibodies and compared to protein standards to determine phenotype. (A) SDS-PAGE separation followed by Coomassie-based staining of haptoglobin standards and proteins in patient sera. The first two lanes from the left contain Hp1-1 and Hp2-2 protein standards, respectively. The third, fourth, and fifth lanes from the left contain study participant sera. As expected in sera, a high abundance of albumin protein was detected in the 66.5 kDa range. (B) Immunoblot of the standards and study participant sera in which haptoglobin proteins are specifically detected by anti-haptoglobin polyclonal antibodies. The first lane from the left contains contains Hp1-1 standard, which has a characteristic band at ~9 kDa representative of the haptoglobin α_1_ chain. The second lane from the left contains Hp2-2 standard, with slower migration bands at higher molecular weights representative of the haptoglobin α_2_ chain. Hp2-2, Hp1-1, and Hp2-1 phenotypes were detected, in the third, fourth, and fifth lanes from the left, respectively.

The details and haptoglobin phenotype of study participants are shown in Table 1. Typical immunoblot analysis of three subjects to determine whether individuals were able to produce zonulin is shown in Fig 1. The majority of study participants were zonulin-producers; 32 of 36 (89%) of the NCWS cohort, 34 of 37 (92%) of patients with untreated coeliac disease, 19 of 20 (95%) patients with ulcerative colitis, and 46 of 49 (94%) healthy individuals had the Hp2-1 or Hp2-2 phenotype (Table 1). The overall haptoglobin phenotype distribution of our 142 study participants was as follows: 11 (8%) were Hp1-1, 84 (59%) were Hp2-1, and 47 (33%) were Hp2-2. These results are in accordance with previous studies that have determined haptoglobin phenotype distributions within study cohorts as well as in the general population [11, 21, 22].

**Table 1:** Demographics and haptoglobin phenotype of study participants.

Subject group	Number of subjects	Mean age, years (SD)	Female sex, n (%)	Haptoglobin phenotype: Hp1-1; Hp2-1; Hp2-2; n (%)
Non-coeliac wheat sensitivity^a^	36	42.6 (12.8)	30 (83)	4 (11); 18 (50); 14 (39)
Coeliac disease^b^	37	36.9 (15.7)	27 (73)	3 (8); 24 (65); 10 (27)
Ulcerative colitis^c^	20	36.9 (11.5)	9 (45)	1 (5); 14 (70); 5 (25)
Healthy	49	39.1 (12.9)	32 (65)	3 (6); 28 (57); 18 (37)

^a^Patients had self-reported, non-coeliac wheat sensitivity and irritable bowel syndrome based on Rome III criteria, and did not have other significant gastrointestinal-related diseases. Coeliac disease was ruled out by the absence of HLA-DQ2 or HLA-DQ8 haplotype or by normal duodenal biopsy [17].

^b^Patients were newly diagnosed and on a gluten-free diet for less than 4 weeks. All had duodenal histology showing a maximum severity of at least Marsh IIIA lesion [18].

^c^Patients were hospitalised with acute severe disease, refractory to intravenous corticosteroid treatment and receiving medical rescue therapy with infliximab [19].

### Serum zonulin levels measured by commercial assay

**Fig 2:**
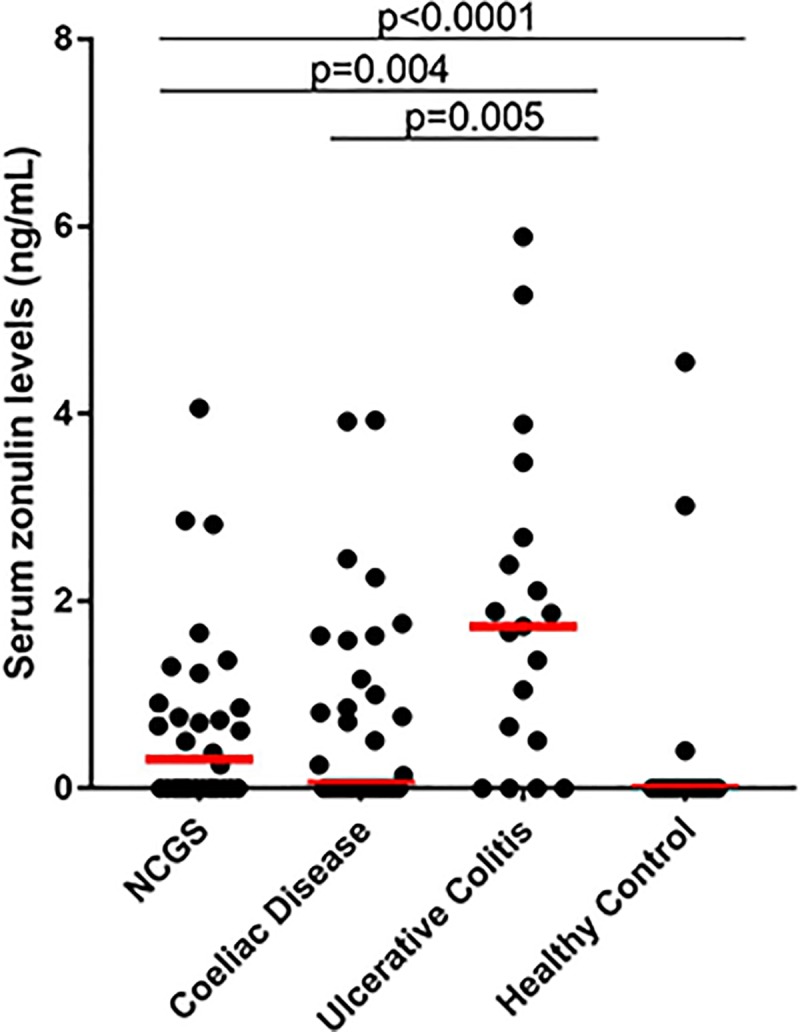
Purported serum zonulin levels (ng/mL) in zonulin producers detected by CUSABIO ELISA assay. Levels of zonulin, as advertised, were determined in non-coeliac wheat sensitivity (NCWS) (n = 36), coeliac disease, (n = 37), and ulcerative colitis (n = 20) patients as well as healthy individuals (n = 49). Compared with the cohort of healthy individuals, patient zonulin levels were elevated (all p<0.0001). Levels in ulcerative colitis were higher than those in NCWS (p = 0.004) and coeliac disease (p = 0.005) with no significant differences between NCWS and coeliac disease. Study samples were ran in duplicate. Red horizontal bars represent median levels for each cohort. Differences in levels between study cohorts were evaluated by Mann-Whitney U tests for nonparametric distributions.

Concentrations of purported zonulin as determined by the CUSABIO assay are shown in Fig 2. Compared with the cohort of healthy individuals with a median (IQR) of 0.00 (0.00) ng/mL, patient median (IQR) values for purported zonulin were elevated (all p<0.0001) at levels of 0.032 (0.90) ng/mL in NCWS, 0.07 (1.27) ng/mL in coeliac disease, and 1.73 (2.17) ng/mL in ulcerative colitis. Levels in ulcerative colitis were higher than those in NCWS (p = 0.004) and coeliac disease (p = 0.005) with no significant differences between NCWS and coeliac disease.

**Fig 3:**
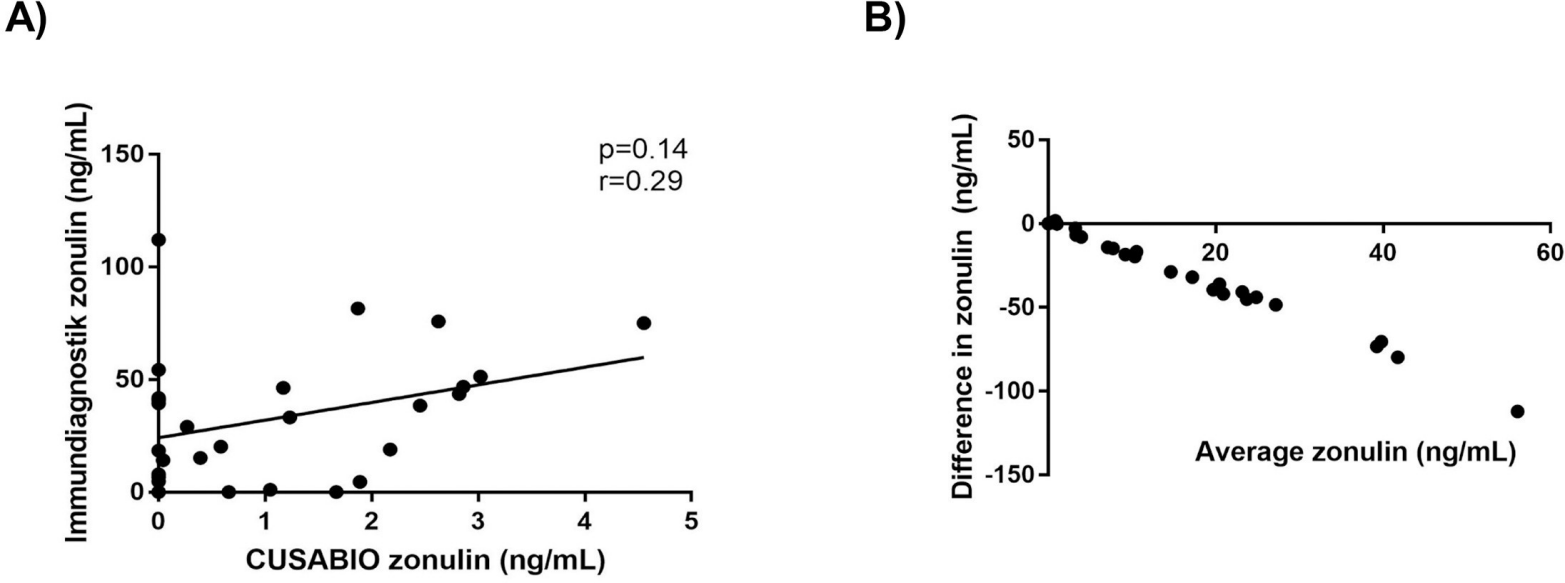
Comparison of purported serum zonulin levels (ng/mL) between CUSABIO and Immundiagnostik ELISA assays. Selected study samples (n = 28) ran in duplicate were compared. (A) Correlation between the two assays (p = 0.14, Spearman’s r = 0.29). (B) Bland-Altman plot calculating difference in zonulin levels vs average of zonulin levels (bias/average discrepancy between methods was -31.62 ng/mL, 95% limits of agreement were from -88.51 to 25.28 ng/mL).

A limited number of patients had serum zonulin measured using the Immundiagnostik assay. There was a poor relationship between the results of the two commercially available assays. No significant correlation between the same samples tested with both assays was observed (n = 28, p = 0.14, r = 0.29; Fig 3A), nor was there significant agreement between the two methods of measurement (bias/average discrepancy between methods was -31.62 ng/mL, 95% limits of agreement were from -88.51 to 25.28 ng/mL; Fig 3B).

Recombinant zonulin as a positive control was not detected by either CUSABIO or Immundiagnostik assay reliably and no significant dose-responses or signals to saturated concentrations were observed. In addition, 2 of 19 participants who were zonulin non-producers had levels detected by CUSABIO assay. Thus, these results cast doubt on the validity of commercially-available zonulin assays to detect the recombinant protein.

### Mass spectrometry analysis of immunoprecipitated proteins and assay standards

The potential identity of serological components captured by both commercial assays was determined by mass spectrometry. A direct comparison approach was used instead of separating immunoprecipitated proteins by SDS-PAGE and excising certain bands of interest to conserve patient serum samples and identify unknown quantities of proteins. The S1 File features full data acquisition ordered in significance from highest to lowest confidence matches. The predominant proteins identified for each immunoprecipitation experiment are summarised in Table 2; major contaminants such as keratin, common background proteins, low confidence single peptide protein matches, and isoforms of matched proteins were excluded.

**Table 2:** Identification of proteins immunoprecipitated from commercial zonulin ELISA assays by LC-MS/MS.

Commercial Assay	Incubation before immunoprecipitation	Complement C3	Haptoglobin	Albumin
CUSABIO	Serum	+	*+*	*+*
	Zonulin	*-*	*+*	*+*
	Negative control	*-*	*-*	*+*
Immundiagnostik	Serum	*+*	*-*	*+*
	Zonulin	*-*	*-*	*+*
	Negative control	*-*	*-*	*+*

+, protein present; -, protein absent.

Incubation with serum followed by immunoprecipitation yielded complement C3, haptoglobin, and albumin as top matches using the CUSABIO assay and complement C3 along with albumin as top matches using the Immundiagnostik assay. Incubation with recombinant zonulin followed by immunoprecipitation yielded haptoglobin and albumin as top matches using the CUSABIO assay and albumin using the Immundiagnostik assay. The negative control for both CUSABIO and Immundiagnostik assays yielded albumin as a top match along with nonspecific proteins.

CUSABIO assay standard, Immundiagnostik tracer, and recombinant zonulin were also analysed by mass spectrometry (S1 File). The most significant and abundant match for CUSABIO assay standard was bovine serum albumin. However, the composition of this product has not been published and, if a recombinant protein was present, the sequence will not have been in the database against which a search can be made. Aside from a collagen alpha protein, there was no strong match in the Immundiagnostik tracer. Similarly, the composition of this product is also unknown and may be a recombinant protein of unknown sequence or a non-protein formulation. The top match for recombinant zonulin was haptoglobin in addition to serum albumin and viral proteins associated with baculovirus expression systems.

### Immunoprecipitation product staining and confirmation of commercial assays’ inability to bind recombinant zonulin/prehaptoglobin-2

**Fig 4:**
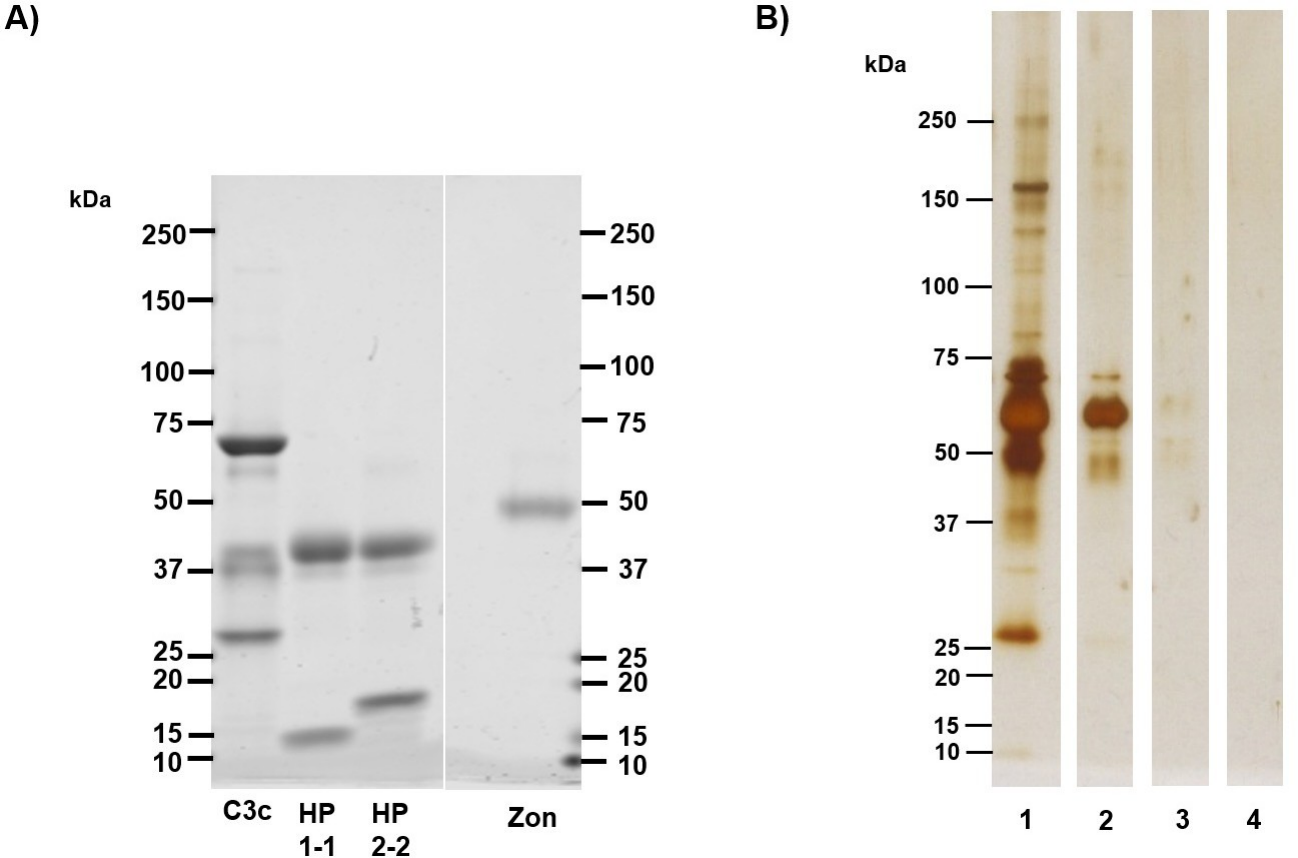
Visualisation of immunoprecipitated protein products and standards. (A) 5 μg of human complement C3c, HP1-1, HP2-2 and recombinant zonulin (zon) standards were separated by SDS-PAGE and stained with Coomassie gel stain. Characteristic bands of standards include the conserved C3c β-chain at 70 kDa, the haptoglobin β-chain at 40 kDa, and the 47 kDa band indicative of recombinant zonulin. (B) Silver staining of immunoprecipitated protein samples detected by LC-MS/MS. Lanes (1) and (2) contain immunoprecipitated proteins from incubation with serum in commercial assays. Lane (1) contains serum proteins captured by CUSABIO assay, which includes complement C3 and haptoglobin as identified by mass spectrometry. Bands at 70 kDa, suggestive of complement C3, and haptoglobin at 40 kDa are present. Lane (2) contains serum proteins captured by Immundiagnostik assay, which includes complement C3 as identified by mass spectrometry. Lanes (3) and (4) contain immunoprecipitated product from incubation of commercial kits with recombinant zonulin. Lane (3), which includes immunoprecipitated product captured by CUSABIO assay, contains trace amounts of protein around the 45–65 kDa range. Mass spectrometry results also indicated a small amount of haptoglobin (S1 File), which remained undetected by silver stain. Lane (4) contained immunoprecipitated product captured by Immundiagnostik assay; no significant detectable proteins aside from albumin were detected by mass spectrometry (S1 File) or indicated by silver stain.

Immunoprecipitated protein products and standards were separated by SDS-PAGE followed by gel staining for visualisation (Fig 4). Protein standards of complement C3c, haptoglobin, and recombinant zonulin each show characteristic bands that were compared visually to immunoprecipitation products. The conserved complement C3 β-chain at approximately 70 kDa, the haptoglobin β-chain at 40 kDa, and the 47 kDa band indicative of recombinant zonulin are clearly indicated by Coomassie stain (Fig 4A). Since the concentration of immunoprecipitation protein products detected by LC-MS/MS was unknown, silver stain was chosen as a more sensitive technique for protein visualisation. The results of staining of the immunoprecipitated protein corresponded to those of the candidate proteins indicated by mass spectrometry. Staining of immunoprecipitated protein products from incubation of the CUSABIO assay with serum revealed distinct bands at 70 kDa, 65 kDa, and 40 kDa, suggestive of the complement C3 β-chain, albumin, and the haptoglobin β-chain, respectively (Fig 4B, Lane 1). The complement C3 β-chain at 70 kDa, albumin at 65 kDa, and faint staining below 50 kDa appeared in the immunoprecipitation sample from incubation of the Immundiagnostik assay with serum (Fig 4B, Lane 2). Addition of recombinant zonulin to assays prior to immunoprecipitation did not yield significant protein products, which confirms the assays’ inability to capture recombinant zonulin (Fig 4B, Lanes 3 and 4). Mass spectrometry analysis for the immunoprecipitated product of CUSABIO capture antibodies incubated with zonulin indicated haptoglobin as a top match, but in trace amounts. Staining of negative control immunoprecipitated products from both commercial assays indicated a wide array of nonspecific proteins, which corresponded to results from mass spectrometry analysis (S1 File).

### Determination of whether candidate proteins detected by mass spectrometry are also detected by CUSABIO assay

We sought to determine whether candidate proteins detected by mass spectrometry (i.e. complement C3 and haptoglobin) are also detected by the CUSABIO assay. We spiked complement C3c, haptoglobin 1–1, and haptoglobin 2–2 standards into the CUSABIO assay and did not observe any dose-responses or reactivity to saturated concentrations.

## Discussion

Due to its putative role in reversible tight junction disassembly, circulating concentration of zonulin has emerged as an increasingly popular biological marker of mucosal barrier integrity. Despite its wide use to assess intestinal mucosal barrier integrity in clinical conditions where a “leaky gut” is suspected, there has been limited information on zonulin levels in patients with gastrointestinal dysfunction. In the current study, sera from well-characterised patient cohorts and controls were used to assess zonulin’s utility as a serological marker of gastrointestinal dysfunction and intestinal mucosal barrier integrity. However, the current commercial assays had significant methodological inconsistencies. Upon further investigation, the assays failed to detect recombinant zonulin/prehaptoglobin-2 protein.

The first indicator of methodological inconsistency involved an inquiry into the assays’ capture antibodies. The epitope to which CUSABIO assay capture antibodies were raised remained unknown and enquiries to the manufacturer remained unanswered (communication with customer service, CUSABIO, Wuhan, China). In contrast, the alternative commercial assay by Immundiagnostik clearly indicated an epitope to which capture antibodies are raised (communication with customer service, Immundiagnostik AG, Bensheim, Germany). This epitope is GGVLVQPG, a peptide sequence synthetically manufactured as AT-1001 or larazotide acetate [6, 23, 24]. Although the purported zonulin receptor has an affinity for this epitope, it remained unclear whether the generation of capture antibodies raised to this sequence would bind to actual circulating zonulin. Another inconsistency was the assays’ apparent detection of zonulin in individuals bearing the Hp1-1 phenotype (i.e. in zonulin non-producers). There was also poor strength of relationship between both commercial assays advertised to detect the same protein (Fig 3). Taken together, these inconsistencies casted initial doubt on the utility of the commercial assays to detect circulating zonulin.

In order to confirm whether these assays are actually detecting zonulin, the recombinant zonulin protein as prehaptoglobin-2, which has been used in principal studies that characterise the protein and demonstrate its ability to decrease transepithelial electrical resistance, was tested in both assays [5]. Dose-responses or signals to high concentrations were not observed; as such, the inability of recombinant zonulin to bind to captured antibodies was confirmed. In support of our ongoing observations, a publication was concurrently released which claimed that the Immundiagnostik assay was not detecting zonulin as advertised, but complement C3 instead [20]. Using an adapted protocol, the captured serum antigens as well as other components from both assays were immunoprecipitated then analysed by mass spectrometry. Our results paralleled those of that publication, as the top match for the Immundiagnostik assay immunoprecipitation product when incubated with serum was complement C3. The serum protein product immunoprecipitated from the CUSABIO assay was best matched with haptoglobin and complement C3. However, addition of recombinant zonulin to both assays did not yield any significant immunoprecipitated protein product as indicated by silver staining (Fig 4B, Lanes 3 and 4). Trace amounts of haptoglobin were detected by mass spectrometry and very slight silver staining was observed for the CUSABIO assay immunoprecipitated product as a result of incubation with recombinant zonulin (S1 File; Fig 4B, Lane 3). Albumin was the only significant mass spectrometry match and no visual staining was observed for the Immundiagnostik assay immunoprecipitated product as a result of incubation with recombinant zonulin (S1 File; Fig 4B, Lane 4). These results confirmed that the current commercial assays are not detecting the protein as advertised and were sufficient evidence to discontinue any further testing of our cohorts and use of our resources.

A follow-up publication by the same group that identified complement C3 as the primary candidate of Immundiagnostik zonulin assay detection took further steps in the characterisation of captured antigens [21]. However, they found that the principal mass spectrometry candidate protein, complement C3, was not detected by the immunoassay. Instead, properdin, a complement-associated and “zonulin-like” protein emerged as the likely candidate of assay detection. Properdin was not detected in our mass spectrometry results for either assay (S1 File) and was a low abundance match in the group’s mass spectrometry results. The group found that Immundiagnostik assay antibodies as well as antibodies raised to recombinant zonulin cross-reacted to this protein, which is of a similar molecular weight (~50 kDa) compared to zonulin. As such, results remain inconclusive, indicating the necessity of additional studies to establish the true identity of target proteins associated with the Immundiagnostik assay, which they assume to be part of the zonulin family.

Our results also hold the same conclusion for the inefficacy of the CUSABIO assay, which does not detect recombinant zonulin. In addition, neither complement C3 nor haptoglobin, despite both being candidate target proteins as determined by mass spectrometry, was detected by the CUSABIO assay. Consequently, we are unable to make valid interpretations of our results with respect to our study cohorts (Fig 2). However, we found this assay to have a different capture antibody than the Immundiagnostik assay, which differs from conclusions made by Scheffler et al. In both primary and follow-up publications, the authors list a table with studies that use the Immundiagnostik assay with a note that “the kit sold by other companies (e.g., ALPCO) is the same as the Immundiagnostik kit” [20, 21]. Our results do not agree with this claim, as there was poor strength of relationship between both commercial assays (Fig 3) and the immunoprecipitation protein products of both assays differed (Fig 4). This indicates distinctive capture antibodies for both commercial assays.

Complement-associated and haptoglobin proteins share similar homology, and both complement C3 and haptoglobin have also been detected together as candidate serological biomarkers by mass spectrometry in an extra-intestinal study [25–27]. The α-chain of haptoglobin contains a complement control protein domain, which is a characteristic component of proteins involved in the regulation of complement (e.g., complement factors H and C1r, mannose-binding lectin-associated serine proteinases and C1 receptor) [26, 28]. Prohaptoglobin is cleaved by complement C1r-like protein, though it does not cleave to the preform of C1s, which is a similar protein to prehaptoglobin-2 [2, 12]. It has been, therefore, hypothesised that the activity of Cr-like protein modulates zonulin production [2]. As such, the close association and shared homology between haptoglobin and complement-associated proteins introduces difficulty in the identification of specific markers by current methods and their roles in the mechanisms of disease. Recent studies show a potential role for complement C3, which is synthesised by murine intestinal epithelial cells, in modulating intestinal barrier integrity [29, 30]. Whether complement C3 acts in synergy with or is independent of the proposed zonulin pathway in the modulation of the intestinal epithelial barrier is an avenue for further study.

In conclusion, the current commercial zonulin ELISA assays investigated in this study detect different proteins, neither of which was zonulin. Therefore, there can be no value of circulating concentrations in assessing intestinal mucosal barrier dysfunction and permeability until the target proteins are indeed identified. The identification of such anomalies should be highlighted to researchers to avoid wasting expenditure of time, money, and precious clinical samples. A monoclonal antibody directed towards human zonulin has been developed for use in western blot analysis, as indicated in the methods of Scheffler et al., though screening zonulin levels by this method of analysis is less quantitative and not preferable for use in the clinical setting compared to an ELISA assay [21]. Commercial ELISA detection methodology may be improved with the development of specific and reliable monoclonal capture and detection antibodies to recombinant zonulin/prehaptoglobin-2 protein. Until assay methodology is improved, we urge the greater scientific and medical community to exercise caution in considering the measurement of serum zonulin as a marker of intestinal barrier dysfunction and permeability.

## Supporting information

S1 Table Plasma or serum CUSABIO and Immundiagnostik commercial assay use in publications. Google Scholar and PubMed search term: “zonulin.” Content was last updated August 7, 2018.
(PDF)

S1 File Full acquisition of mass spectrometry data for assay standards and each immunoprecipitation experiments described. Spreadsheet tabs indicate specific assay standard or immunoprecipitation experiment.
(XLSX)
