# Supplementary material for: Serum zonulin as a marker of intestinal mucosal barrier function: May not be what it seems
Source: PLoS One. 2019 Jan 14;14(1):e0210728. doi: 10.1371/journal.pone.0210728 (PMC6331146; doi:10.1371/journal.pone.0210728)
Supplement: S1 Table — Google Scholar and PubMed search term: “zonulin.” Content was last updated August 7, 2018. (PDF) [file pone.0210728.s001.pdf]

S1 Table. Plasma or serum CUSABIO and Immundiagnostik commercial assay use in publications. Google Scholar and PubMed search term: “zonulin.” Content was last updated August 7, 2018.

Assay	Author (Year)	Title	Journal
CUSABIO	Çakir M et al. (2017)	Effects of long-term symbiotic supplementation in addition to lifestyle changes in children with obesity-related non-alcoholic fatty liver disease.	The Turkish Journal of Gastroenterology
CUSABIO	Dong S et al. (2018)	Protective effect of 1, 25-dihydroxy vitamin D3 on pepsin-trypsin-resistant gliadin-induced tight junction injuries.	Digestive Diseases and Sciences
CUSABIO	Feuerecker M et al. (2016)	Headache under simulated microgravity is related to endocrine, fluid distribution, and tight junction changes.	Pain
CUSABIO	Greis C et al. (2017)	Intestinal T lymphocyte homing is associated with gastric emptying and epithelial barrier function in critically ill: a prospective observational study.	Critical Care
CUSABIO	Jayashree B et al. (2013)	Increased circulatory levels of lipopolysaccharide (LPS) and zonulin signify novel biomarkers of proinflammation in patients with type 2 diabetes.	Molecular and Cellular Biochemistry

CUSABIO	Küme T et al. (2017)	The relationship between serum zonulin level and clinical and laboratory parameters of childhood obesity.	Journal of Clinical Research in Pediatric Endocrinology
CUSABIO	Küme T et al. (2017)	The relationship between serum zonulin level and clinical and laboratory parameters of childhood obesity.	Journal of Clinical Research in Pediatric Endocrinology
CUSABIO	Mall JPG et al. (2018)	Are self-reported gastrointestinal symptoms among older adults associated with increased intestinal permeability and psychological distress?	BMC Geriatrics
CUSABIO	Ramos CI et al. (2018)	Effect of prebiotic (fructooligosaccharide) on uremic toxins of chronic kidney disease patients: a randomized controlled trial.	Nephrology Dialysis Transplantation
CUSABIO	Zhang D et al. (2014)	Circulating zonulin levels in newly diagnosed Chinese type 2 diabetes patients.	Diabetes Research and Clinical Practice
Immundiagnostik	Barceló A et al. (2016)	Gut epithelial barrier markers in patients with obstructive sleep apnea.	Sleep Medicine
Immundiagnostik	Carrera-Bastos P et al. (2018)	Serum zonulin and endotoxin levels in exceptional longevity versus precocious myocardial infarction.	Aging and Disease
Immundiagnostik	Chuanwei L et al. (2016)	Zonulin regulates intestinal permeability and facilitates enteric bacteria permeation in coronary artery disease.	Scientific Reports

Immundiagnostik	Chwist A et al. (2014)	A composite model including visfatin, tissue polypeptide-specific antigen, hyaluronic acid, and hematological variables for the diagnosis of moderate-to-severe fibrosis in non-alcoholic fatty liver disease: a preliminary study.	Polish Archives of Internal Medicine
Immundiagnostik	Damms-Machado A et al. (2017)	Gut permeability is related to body weight, fatty liver disease, and insulin resistance in obese individuals undergoing weight reduction.	The American Journal of Clinical Nutrition
Immundiagnostik	de Roos et al. (2017)	The effects of a multispecies probiotic on migraine and markers of intestinal permeability – results of a randomized placebo-controlled study.	European Journal of Clinical Nutrition
Immundiagnostik	Ficek J et al. (2017)	Relationship between plasma levels of zonulin, bacterial lipopolysaccharides, D-lactate and markers of inflammation in haemodialysis patients.	International Urology and Nephrology
Immundiagnostik	Gentser L et al. (2018)	Increased jejunal permeability in human obesity is revealed by a lipid challenge and is linked to inflammation and type 2 diabetes.	The Journal of Pathology
Immundiagnostik	Gerdes S et al. (2011)	Zonulin may not be a marker of autoimmunity in patients with psoriasis.	Acta Dermato Venereologica
Immundiagnostik	Houttu N et al. (2017)	Overweight and obesity status in pregnant women are related to intestinal microbiota and serum metabolic and inflammatory profiles	Clinical Nutrition

Immundiagnostik	Hunt P et al. (2014)	Gut epithelial barrier dysfunction and innate immune activation predict mortality in treated HIV infection.	The Journal of Infectious Diseases
Immundiagnostik	Józefczuk J et al. (2018)	The occurrence of antibodies against gluten in children with autism spectrum disorders does not correlate with serological markers of impaired intestinal permeability.	Journal of Medicinal Food
Immundiagnostik	Karhu E et al. (2017)	Exercise and gastrointestinal symptoms: running-induced changes in intestinal permeability markers of gastrointestinal function in asymptomatic and symptomatic runners.	European Journal of Applied Physiology
Immundiagnostik	Kim AS et al. (2018)	Plasma concentrations of zonulin are elevated in obese men with fatty liver disease.	Diabetes, Metabolic Syndrome and Obesity: Targets and Therapy
Immundiagnostik	Kim JH et al. (2018)	Zonulin level, a marker of intestinal permeability, is increased in association with liver enzymes in young adolescents.	Clinica Chimica Acta
Immundiagnostik	Kim S et al. (2018)	Imbalance of gut microbiome and intestinal epithelial barrier dysfunction in patients with high blood pressure.	Clinical Science
Immundiagnostik	Klaus DA et al. (2013)	Increased plasma zonulin levels in patients with sepsis.	Biochemica Medica (Zagreb)

Immundiagnostik	Koay WLA et al. (2018)	Intestinal integrity biomarkers in early antiretroviral-treated perinatally HIV-1-intected infants.	The Journal of Infectious Diseases
Immundiagnostik	Kvehaugen AS et al. (2018)	Is perceived intolerance to milk and wheat associated with the corresponding IgG and IgA food antibodies? A cross sectional study in subjects with morbid obesity and gastrointestinal symptoms.	BMC Gastroenterology
Immundiagnostik	Lamprecht M et al. (2015)	Effects of zeolite supplementation on parameters of intestinal barrier integrity, inflammation, redoxbiology and performance in aerobically trained subjects.	Journal of the International Society of Sports Nutrition
Immundiagnostik	Li C et al. (2016)	Zonulin regulates intestinal permeability and facilitates enteric bacteria permeation in coronary artery disease.	Scientific Reports
Immundiagnostik	Łukaszyk E et al. (2018)	Zonulin, inflammation and iron status in patients with early stages of chronic kidney disease.	International Urology and Nephrology
Immundiagnostik	Łukaszyk M et al. (2015)	Iron status and inflammation in early stages of chronic kidney disease.	Kidney and Blood Pressure Research
Immundiagnostik	Malyszko et al. (2014)	Zonulin, iron status, and anemia in kidney transplant recepients: are they related?	Transplantation Proceedings

Immundiagnostik	Mokkala K et al. (2016)	Gut microbiota richness and composition and dietary intake of overweight pregnant women are related to serum zonulin concentration, a marker for intestinal permeability.	The Journal of Nutrition
Immundiagnostik	Mokkala K et al. (2017)	Evaluation of serum zonulin for use as an early predictor for gestational diabetes.	Nutrition and Diabetes
Immundiagnostik	Mokkala K et al. (2017)	Increased intestinal permeability, measured by serum zonulin, is associated with metabolic risk markers in overweight pregnant women.	Metabolism
Immundiagnostik	Mokkala K et al. (2018)	The impact of probiotics and n-3 long-chain polyunsaturated fatty acids on intestinal permeability in pregnancy: a randomised clinical trial.	Beneficial Microbes
Immundiagnostik	Mondal D et al. (2012)	Contribution of enteric infection, altered intestinal barrier function, and maternal malnutrition to infant malnutrition in Bangladesh.	Clinical Infectious Diseases
Immundiagnostik	Moreno-Navarrete JM et al. (2012)	Circulating zonulin, a marker of intestinal permeability, is increased in association with obesity-associated insulin resistance.	PLoS One
Immundiagnostik	Mörkl S et al. (2018)	Gut microbiota, dietary intakes and intestinal permeability reflected by serum zonulin in women.	European Journal of Nutrition

Immundiagnostik	Muñoz-Cano R et al. (2017)	Distinct transcriptome profiles differentiate NSAID-dependent from NSAID-independent food anaphylaxis.	The Journal of Allergy and Clinical Immunology
Immundiagnostik	Mwape I et al. (2017)	Immunogenicity of rotavirus vaccine (Rotarix^{TM}) in infants with environmental enteric dysfunction.	PLoS One
Immundiagnostik	Ohlsson B et al. (2016)	Two meals with different carbohydrate, fat and protein contents render equivalent postprandial plasma levels of calprotectin, cortisol, tryglycerides and zonulin.	International Journal of Food Sciences and Nutrition
Immundiagnostik	Ohlsson B et al. (2017)	Calprotectin in serum and zonulin in serum and feces are elevated after introduction of a diet with lower carbohydrate content and higher fiber, fat and protein contents.	Biomedical Reports
Immundiagnostik	Ohlsson B et al. (2017)	Higher levels of zonulin may rather be associated with increased risk of obesity and hyperlipidemia, than with gastrointestinal symptoms or disease manifestations.	International Journal of Molecular Sciences
Immundiagnostik	Orlando A et al. (2014)	Lactobacillus GG restoration of the gliadin induced epithelial barrier disruption: the role of cellular polyamines.	BMC Microbiology
Immundiagnostik	Ott B et al. (2017)	Effect of caloric restriction on gut permeability, inflammation markers, and fecal microbiota in obese women.	Scientific Reports

Immundiagnostik	Ott B et al. (2018)	Short-term overfeeding with dairy cream does not modify gut permeability, the fecal microbiota, or glucose metabolism in young healthy men.	The Journal of Nutrition
Immundiagnostik	Pacifico L et al. (2014)	Increased circulating zonulin in children with biopsy-proven non-alcoholic fatty liver disease.	World Journal of Gastroenterology
Immundiagnostik	Pacifico L et al. (2014)	Increased circulating zonulin in children with biopsy-proven non-alcoholic fatty liver disease.	World Journal of Gastroenterology
Immundiagnostik	Pärtty A et al. (2017)	Infantile colic is associated with low-grade systemic inflammation.	Journal of Pediatric Gastroenterology and Nutrition
Immundiagnostik	Pei R et al. (2017)	Low-fat yogurt consumption reduces biomarkers of chronic inflammation and inhibits markers of endotoxin exposure in healthy premenopausal women: a randomised controlled trial.	British Journal of Nutrition
Immundiagnostik	Qi Y et al. (2017)	Intestinal permeability biomarker zonulin is elevated in healthy aging.	Journal of the American Medical Directors Association
Immundiagnostik	Rees D et al. (2018)	A randomised, double-blind, cross-over trial to evaluate bread, in which gluten has been pre-digested by prolyl endoprotease treatment, in subjects self-reporting benefits of adopting a gluten-free or low-gluten diet.	British Journal of Nutrition

Immundiagnostik	Ruiz-Núñez B et al. (2018)	Higher prevalence of “low T3 syndrome” in patients with chronic fatigue syndrome: a case-control study.	Frontiers in Endocrinology (Lausanne)
Immundiagnostik	Russo F et al. (2012)	Inulin-enriched pasta improves intestinal permeability and modifies the circulating levels of zonulin and glucagon-like peptide 2 in healthy young volunteers.	Nutrition Research
Immundiagnostik	Russo F et al. (2013)	The effects of fluorouracil, epirubicin, and cyclophosphamide (FEC60) on the intestinal barrier function and gut peptides in breast cancer patients: an observational study.	BMC Cancer
Immundiagnostik	Sanches-Alcoholado L et al. (2017)	Role of gut microbiota on cardio-metabolic parameters and immunity in coronary artery disease patients with and without type-2 diabetes mellitus.	Frontiers in Microbiology
Immundiagnostik	Scorletti E et al. (2018)	Design and rationale of the INSYTE study: A randomised, placebo controlled study to test the efficacy of a symbiotic on liver fat, disease biomarkers and intestinal microbiota in non-alcoholic fatty liver disease.	Contemporary Clinical Trials
Immundiagnostik	Sheen YH et al. (2018)	Serum zonulin is associated with presence and severity of atopic dermatitis in children, independent of total IgE and eosinophil.	Clinical and Experimental Allergy

Immundiagnostik	Šket R et al. (2017)	Hypoxia and inactivity related physiological changes (constipation, inflammation) are not reflected at the level of gut metabolites and butyrate producing microbial community; the PlanHab Study.	Frontiers in Physiology
Immundiagnostik	Stadlbauer V et al. (2015)	Lactobacillus casei Shirota supplementation does not restore gut microbiota composition and gut barrier in metabolic syndrome: a randomized pilot study.	PLoS One
Immundiagnostik	Stenman LK et al. (2016)	Probiotic with or without fiber controls body fat mass, associated with serum zonulin, in overweight and obese adults – randomized controlled trial.	EBioMedicine
Immundiagnostik	Stevens BR et al. (2018)	Increased human intestinal barrier permeability plasma and FABP2 correlated with plasma LPS and altered gut microbiome in anxiety and depression.	Gut
Immundiagnostik	Strewe C et al. (2018)	PlanHab Study: Consequences of combined normobaric hypoxia and bed rest on adenosine kinetics.	Scientific Reports
Immundiagnostik	Tarko A et al. (2017)	Zonulin: a potential marker of intestinal injury in newborns.	Disease Markers

Immundiagnostik	Wilms E et al. (2016)	Effects of supplementation of the Synbiotic Ecologic® 825/FOS P6 on intestinal barrier function in healthy humans: a randomized control trial.	PLoS One
Immundiagnostik	Wosiewicz P et al. (2016)	Portal vein thrombosis in cirrhosis is not associated with intestinal barrier disruption or increased platelet aggregability.	Clinics and Research in Hepatology and Gastroenterology
Immundiagnostik	Żak-Gołab A et al. (2013)	Gut microbiota, microinflammation, metabolic profile and zonulin concentration in obese and normal weight subjects.	International Journal of Endocrinology
Immundiagnostik	Zhang D et al. (2015)	Serum zonulin is elevated in women with polycystic ovary syndrome and correlates with insulin resistance and severity of anovulation.	European Journal of Endocrinology
